# Examining the relationships between students’ perceptions of technology, pedagogy, and cognition: the case of immersive virtual reality mini games to foster computational thinking in higher education

**DOI:** 10.1186/s40561-023-00233-1

**Published:** 2023-02-23

**Authors:** Friday Joseph Agbo, Sunday Adewale Olaleye, Matt Bower, Solomon Sunday Oyelere

**Affiliations:** 1 School of Computing, University of Eastern Finland, P.O. Box 111, FIN-80101 Joensuu, Finland; 2 Computing and Data Science, Willamette University, Salem, OR USA; 3 School of Business, Jamk University of Applied Sciences, Rajakatu 35, 40100 Jyvaskyla, Finland; 4 School of Education, Macquarie University, Sydney, NSW Australia; 5 Department of Computer Science, Electrical and Space Engineering, Luleå University of Technology, 93187 Skellefteå, Sweden

**Keywords:** Computational thinking, Immersive virtual reality, Mini games, Cognition, Reflective thinking, Technology-mediated learning, Higher education

## Abstract

Researchers are increasingly exploring educational games in immersive virtual reality (IVR) environments to facilitate students’ learning experiences. Mainly, the effect of IVR on learning outcomes has been the focus. However, far too little attention has been paid to the influence of game elements and IVR features on learners’ perceived cognition. This study examined the relationship between game elements (challenge, goal clarity, and feedback) as pedagogical approach, features of IVR technology (immersion and interaction), and learners’ perceived cognition (reflective thinking and comprehension). An experiment was conducted with 49 undergraduate students who played an IVR game-based application (iThinkSmart) containing mini games developed to facilitate learners’ computational thinking competency. The study employed partial least squares structural equation modelling to investigate the effect of educational game elements and learning contents on learner’s cognition. Findings show that goal clarity is the main predictor of learners’ reflective thinking and comprehension in an educational game-based IVR application. It was also confirmed that immersion and interaction experience impact learner’s comprehension. Notably, adequate learning content in terms of the organisation and relevance of the content contained in an IVR game-based application significantly moderate learners’ reflective thinking and comprehension. The findings of this study have implications for educators and developers of IVR game-based intervention to facilitate learning in the higher education context. In particular, the implication of this study touches on the aspect of learners’ cognitive factors that aim to produce 21st-century problem-solving skills through critical thinking.

## Introduction

Due to how affordable and portable technology has become, immersive virtual reality (IVR) technology is mediating teaching and learning in several educational contexts (Bower and Jong, 2020). IVR technology does not only create an opportunity to supplement teaching and learning but also allows students to interact with learning objects. Besides, the features of IVR technology—immersion, interaction, presence, and immediacy—contribute to its affordances in education (Makransky et al., 2019). Indeed, IVR technology is used for training and simulation of educational concepts (Lui et al., 2020), which makes it possible to represent almost everything as a learning object in a virtual environment for learners to gain experiential knowledge (Alrehaili & Al Osman, 2019).

Computational thinking (CT) is a growing field that demonstrates a pedagogical approach to fostering critical thinking and problem-solving skills among contemporary learners (Wing, 2009; Carretero et al., 2017). CT involves thought processes to solve a given problem. CT includes concepts such as algorithmic thinking, problem decomposition, problem abstraction, pattern recognition, and recursive thinking (Agbo et al., 2021b). The introduction of these concepts into schools’ curriculum allows young learners to have digital literacy and develop thinking skills that can foster their ability to computationally solve real-world problems (Aho, 2012). Developing CT competency is essential for K-12 (Wing, 2009) and higher education students (Cachero et al., 2020). Hence, the integration of CT in the higher education context is currently gaining ground nowadays (de Jong & Jeuring, 2020). Studies have shown how university students who do not have a computer programming background found programming courses difficult to understand (Agbo et al., 2019; Gamage, 2021; Liu & Zhong, 2018). This difficulty could be due to their lack of comprehension of the concepts of programming. One way to reduce the difficulty in understanding computer programming is to develop their CT knowledge. This knowledge of CT will support students’ creative thinking skills that can be applied to problem-solving (Sukirman et al., 2021).

To develop a state-of-the-art technology-based intervention to facilitate 21st-century skills including CT, IVR and game-based learning (GBL) is a viable approach. Studies have shown that educational IVR mini games can impact learners’ cognition and learning outcomes in creative thinking, reflective thinking, and self-efficacy (Lee et al., 2010; Makransky & Petersen, 2019; Kim et al., 2020; Agbo et al., 2022). Despite these positive outcomes from previous studies, there are concerns that may affect the use and adoption of IVR in education. For example, it has been reported that IVR technology could lead to a higher cognitive load, more distractions to learners, and poorer performance (Makransky et al., 2019). To address these concerns, there is a need to examine the relationship between IVR features and educational game elements on learners’ cognition. In other words, to reinforce the affordances of VR and GBL for a 21st-century learning experience, there must be a concerted effort by scholars to examine critical elements of both the technology and pedagogy using their characteristic elements. In addition, investigating the affordances of IVR and GBL vis-à-vis learner’s cognitive benefit is a continuing concern among educators in the higher education context. Therefore, game elements which include challenges, goal clarity, feedback, and adequacy of learning content, and IVR features such as immersion and interaction form the study’s variables being examined. Although studies have investigated the impact of IVR features on learning outcomes (Lin et al., 2020; Barrett et al., 2021), the effect of IVR and game elements on learners’ cognition is inadequately researched (Imlig-Iten & Petko, 2018). Hence, this exploratory study builds on the path model of Hamari et al. (2016) and Lin et al. (2020) to examine how features of IVR and game elements of an educational tool influence learners’ reflective thinking and cognition.

## Theoretical foundation

### Technology-mediated learning theory

According to literature, technology-mediated learning (TML) refers to an environment in which learners interact with instructional materials, peers, and or instructors where information technology plays an intermediary role in connecting the different stakeholders within a learning situation (Alavi, 1994; Ryoo & Lee, 2016). Essentially, TML theoretical assumption stresses that in educational technology research, technologies themselves have no intentions but are rather featured to convey meanings between stakeholders in a learning environment (Lin et al., 2020). In evaluating the effect of a learning environment within the theoretical framework of TML, Bower (2019) has provided several scenarios to guide researchers. For example,

Does technology-mediated learning theory apply in computer-assisted learning situations where a program of instruction or software package has been written for use by a student in isolation at a stand-alone machine? Even though such learning may not occur in a social context, the digital content has been composed by educators and is mediated using technology, so technology-mediated learning theory may be useful to examine and explain effects. (Bower, 2019, p. 1043)

Nowadays, many studies are carried out to examine how teaching and learning are being mediated by IVR applications to facilitate students’ learning experience in terms of cognition (Cheng & Tsai, 2020), creative thinking, and reflective thinking (Makransky & Petersen, 2019). For example, Lin et al. (2020) recently investigated the effect of IVR application on learning outcomes using a two-path model. According to Lin and colleagues, when designing a TML intervention, technological features of VR, such as immersion, should be well designed since it strongly predicts learners’ motivation and learning effectiveness. Additionally, Cheng and Tsai (2020) investigated the effect of student learning traits mediated with immersive features of IVR on their learning attitude. Findings from Cheng and colleague suggests that students’ intrinsic value and self-regulation for learning could positively impact their sense of immersion.

### Immersive virtual reality and mini games in education

IVR has varied applications in education and training. Prior research on the effect of IVR on learner’s performance exists (Agbo et al., 2021a). For instance, Makransky et al. (2019) investigated the effectiveness of an IVR application in promoting science education compared to video-based instructions. In their study, Makransky and colleagues revealed that IVR provides more interactivity, which in turn is beneficial to learning. Petersen et al. (2020) conducted a study that demonstrated how learners could travel virtually to relevant sites to learn about climate while in physical classrooms. According to Petersen and his colleagues, IVR application increases students’ interest, knowledge, and self-efficacy, while pretraining affects cognitive load, affecting learners’ performance.

Similarly, Lui et al. (2020) recently explored how to teach complex scientific concepts such as gene regulation through simulation within an IVR environment. Their experiment revealed that students who learned the concepts using the IVR approach gained better knowledge compared to those who did not learn using the IVR intervention but the traditional approach. While previous research underscores the relevance of IVR for teaching and learning, this current study investigates how students think IVR game-based application can facilitate their computational thinking by exploring the relationships between IVR features and game elements on learners’ perceived cognition.

Furthermore, educational mini games developed as an IVR application is a good approach to foster higher-order thinking skills where learners are not only motivated to learn but also engaged through game challenges that start easily and then progressively become difficult (Chaves et al., 2021; Hamari et al., 2016). Educational mini games are short types of serious games that are developed to be playable independently to gain micro knowledge (Agbo et al., 2021b). Unlike serious games, mini games are flexible, simple, and easy to learn. The benefits inherent in mini games create the opportunity to achieve a small unit of learning objectives; hence, this study conducted an experiment with IVR mini games using low-cost head-mounted displays (HMD) and hand controllers. Studies have shown how the use of IVR applications with low-cost HMD such as Google Cardboard and a Bluetooth hand controller are increasingly deployed for instructional practices and science education (Parong & Mayer, 2018; Cheng & Tsai, 2020; Agbo et al., 2022).

### Computational thinking in higher education

Computational thinking was made popular by Wing in her presentation at the Communications of the ACM (Association for Computing Machinery) anniversary celebration (Wing, 2006). Since then, teaching and learning of computational thinking have been integrated into the school curriculum, mostly in K-12 settings (Grover & Pea, 2013). Several debatable topics have emerged over the years regarding, for example, how computational thinking should be taught at school (Mannila et al., 2014), who should be taught computational thinking (Lockwood & Mooney, 2018), what common grounds should be developed for computational thinking and computing education, and whether computational thinking should be integrated into higher education institutions’ curriculum (Hu, 2011; Czerkawski & Lyman, 2015; Apiola & Sutinen, 2021).

A systematic review study revealed that teaching computational thinking in higher education institutions began in 2010, where educators designed courses to infuse computational thinking concepts into their classes (Agbo et al., 2019). Furthermore, de Jong and Jeuring (2020) presented a scoping literature review of tools and interventions used to teach computational thinking in higher education and how effective these interventions are. Their findings revealed that varied disciplines, including information systems, journalism, sociology, tourism, and even engineering, have discussed or implemented some computational thinking concepts. However, programming education classes were the most likely subject area to integrate computational thinking concepts (de Jong & Jeuring, 2020). By “computational thinking concepts," we mean topics focusing on problem-solving skills, algorithmic thinking, problem decomposition, pattern recognition, problem abstraction, and recursive thinking.

Integration of computational thinking in higher education to facilitate programming education is on the rise (de Jong & Jeuring, 2020). Educators are exploring more contemporary approaches to using technology in learning and teaching. That is why we need to investigate how to do it well. Besides, the affordability of advanced technology and smart systems such as cloud computing, the internet of things, smartphones, and wearables, has caused education to be more flexible, autonomous, and ubiquitous, such that learners can gain more benefits of enhanced learning experience that is engaging and motivating (Lin et al., 2020). Hence, leveraging a GBL approach and virtual reality (VR) as state-of-the-art technology can provide an opportunity for an enhanced learning experience (Alrehaili & Al Osman, 2019) for learners in the higher education context.

### Development of the research hypothesis

**Fig. 1:**
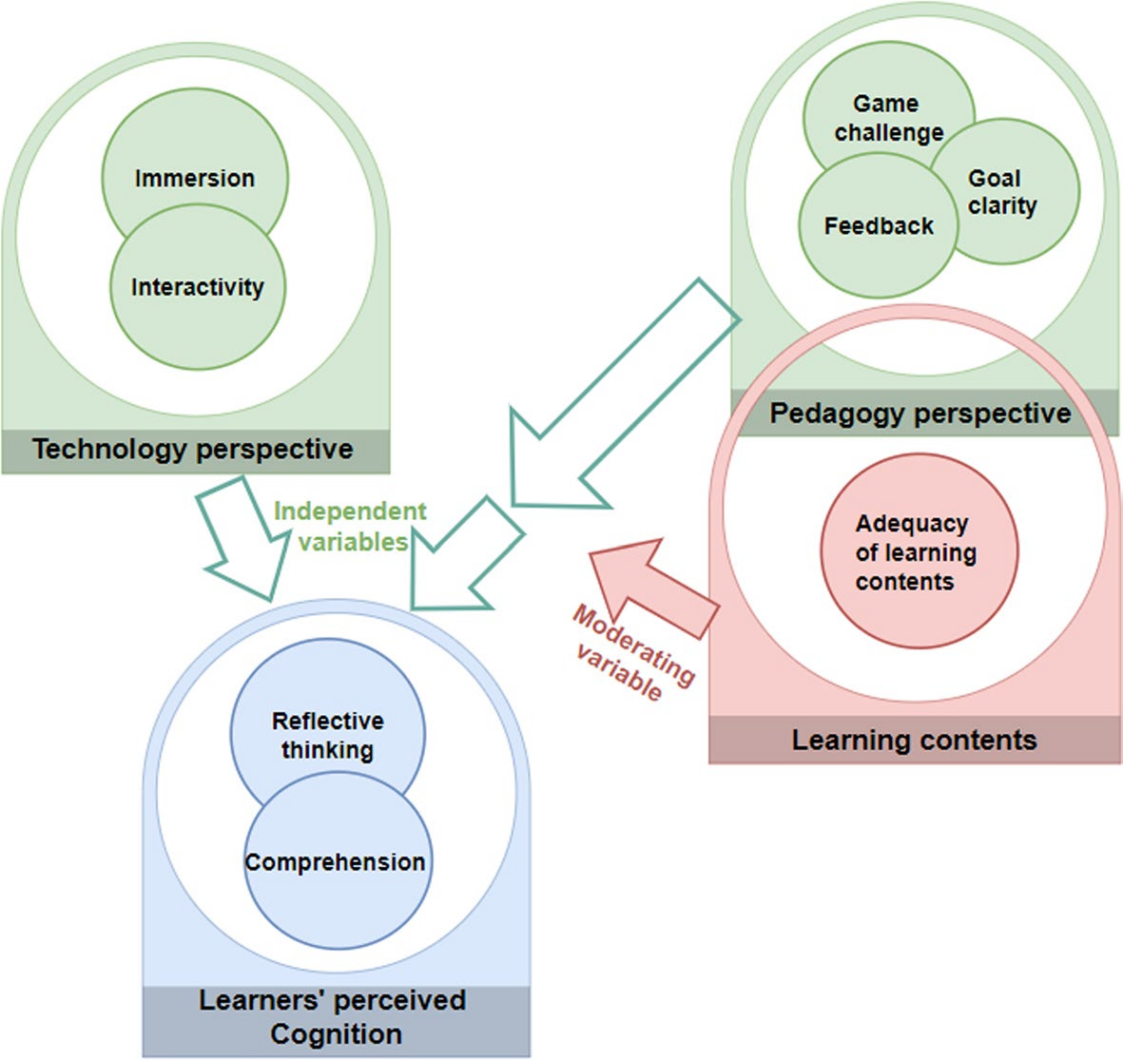
Conceptual framework of the relationships between IVR, game-based TML, and perceived learners’ cognition

The relationships between the dependent and independent variables for this study were motivated by the path models of Hamari et al. (2016) and Lin et al. (2020) to develop the game element constructs, IVR technology constructs, and cognition constructs. As shown in the conceptual framework of the relationships between IVR, GBL, and perceived learners’ cognition (Fig. 1), two components, including the technology (immersion and interactivity) and pedagogy (game challenge, goal clarity, and feedback), form the independent variables. The cognition component constitutes the dependent variables (reflective thinking and comprehension), whereas learning content is the moderating variable.

**Fig. 2:**
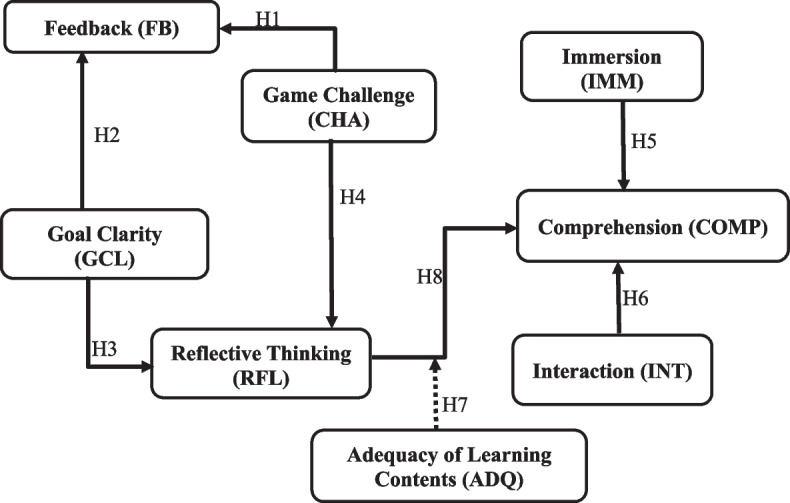
Structural model showing the interrelationship among the variables

Within the technology component, immersion, and interaction (Makransky & Petersen, 2019) are the constructs investigated. Whereas, in the pedagogy component, game elements such as challenge, goal clarity, and feedback (Fokides et al., 2019; Fu et al., 2009) were the underlying constructs. Figure 2 depicts the structural model for this study, which seeks to examine the relationships between the constructs and how they influence the learner’s perceived cognition.

### Effect of educational game elements on learner’s perceived cognition

According to Plass et al. (2015), game elements are critical factors that determine the learning outcome of an educational game. They posit that for any game research, cognitive engagement is one of the outcomes of educational GBL, influenced by the three key elements of almost all games (i.e., challenge, response, and feedback). Feedback is a major element of a game that allows the player to be aware of the current level of achievement, determine the gap between the current stage and the expected goal, and provide relevant tips required to complete the goal (Fu et al., 2009). Players of a game can receive feedback in form of rewards, warnings, hints, or instructions. The appropriateness and timeliness of feedback provided to players of an educational game could influence the perceived level of challenge. According to Beghetto (2018), Challenge "literarily means an invitation or call to action" (p. 13). In game design, elements such as challenges can foster players’ curiosity leading to intrinsic motivation (Plass et al., 2015).

Nevertheless, an adequate level of challenge with goal clarity is required to maintain a state of flow for an effortless learning experience. Goal clarity defines the intermediate and primary objectives of an educational game (Wang et al., 2014). According to the literature, educational game challenge and goal clarity significantly impact learners' cognitive processing (Mayer, 2014), human behaviours, and learning outcomes (Fokides et al. (2019); Wang et al., 2014. Therefore, the kind of challenge perceived by a player can determine what feedback could be appropriate to overcome such challenge; hence, this study hypothesized that:

#### H1

Game challenge will positively influence the feedback received when playing VR mini games.

#### H2

Goal clarity will positively influence the feedback received from playing VR mini games.

### Impact of learning contents of IVR mini games on learners’ perceived cognition

The central focus of any technology-mediated learning intervention is usually to enhance the learning experience with the aim of improving knowledge. Therefore, cognition in this context indicates the knowledge and capability gained after undergoing a learning process using information technology to mediate between the learner and the learning contents. The knowledge gained can be applied to solving difficult tasks. In this study, learners' perceived cognition refers to participants' self-ratings of cognitive benefits rather than measures of learning or performance. Learners' perceived cognition (reflective thinking and comprehension) has been adapted from Lin et al. (2020) model of the TML framework in the VR context. According to the literature, comprehension is a fundamental element of the cognitive learning process whose impact on learners in an IVR learning environment is explored (Zhao et al., 2020). Similarly, this study conceptualizes reflective thinking as the state of mind where learners engage in self-inquiry to understand a certain phenomenon and clarify possible doubts (Makransky & Petersen, 2019). According to Chen et al. (2019), reflective thinking is a complex skill that would require critical thinking and problem-solving skills. Based on this discussion, this study hypothesized that:

#### H3

Goal clarity will have a positive effect on learners’ reflective thinking when playing VR mini games.

#### H4

Game challenge will positively influence learners’ reflective thinking when playing VR mini games.

### Important features of IVR on learner’s perceived cognition

As stated earlier, research has shown that IVR features which include immersion, interaction, the immediacy of control, and representational fidelity could influence learning outcomes (Lee et al., 2010; Lin et al., 2020). Immersion is a characteristic of VR technology that creates the opportunity for a user to perceive of being in a real environment yet in a virtual environment. Although the degree of immersion experienced by users of VR is subject to variation, the technological capabilities of VR devices, the sensors, screen resolutions, and even the virtual contents have a great impact on the way the technology influences user's immersion (Mütterlein, 2018). Similarly, interaction within a virtual environment is possible through the integration of different devices such as HMDs, motion sensors, and hand controllers of varied interaction capabilities. The more a technology-mediated learning tool allows for interaction between the learners, learning contents, and other objects within the virtual environment, the more positively impactful the learning experience becomes. Based on the discussion, this study proposed the following hypotheses.

#### H5

Immersion will have a positive effect on learners’ comprehension when playing VR mini games.

#### H6

The interaction will have a positive effect on learners' comprehension when playing VR mini games.

#### H7

Reflective thinking predicts learners’ comprehension when playing VR mini games.

In the context of this study, the concept of learning content in relationship to TML refers to lesson topics, lessons, and concepts to be learnt including learning materials, instructions, and tutorials provided in IVR application to facilitate learners' comprehension. According to Lee et al. (2020), the content quality of a VR application generally refers to the appropriateness, accuracy, flow of the presentation, and completeness of learning materials. Therefore, the adequacy of learning content could moderate the learning outcome by enhancing the degree of reflective thinking a learner may possess. Therefore, this study proposed the following hypothesis.

H8. Adequacy of learning contents will positively moderate reflective thinking and can influence learners’ comprehension when playing VR mini games.

## Methods

### Description of the VR application and the mini games to foster computational thinking

**Fig. 3:**
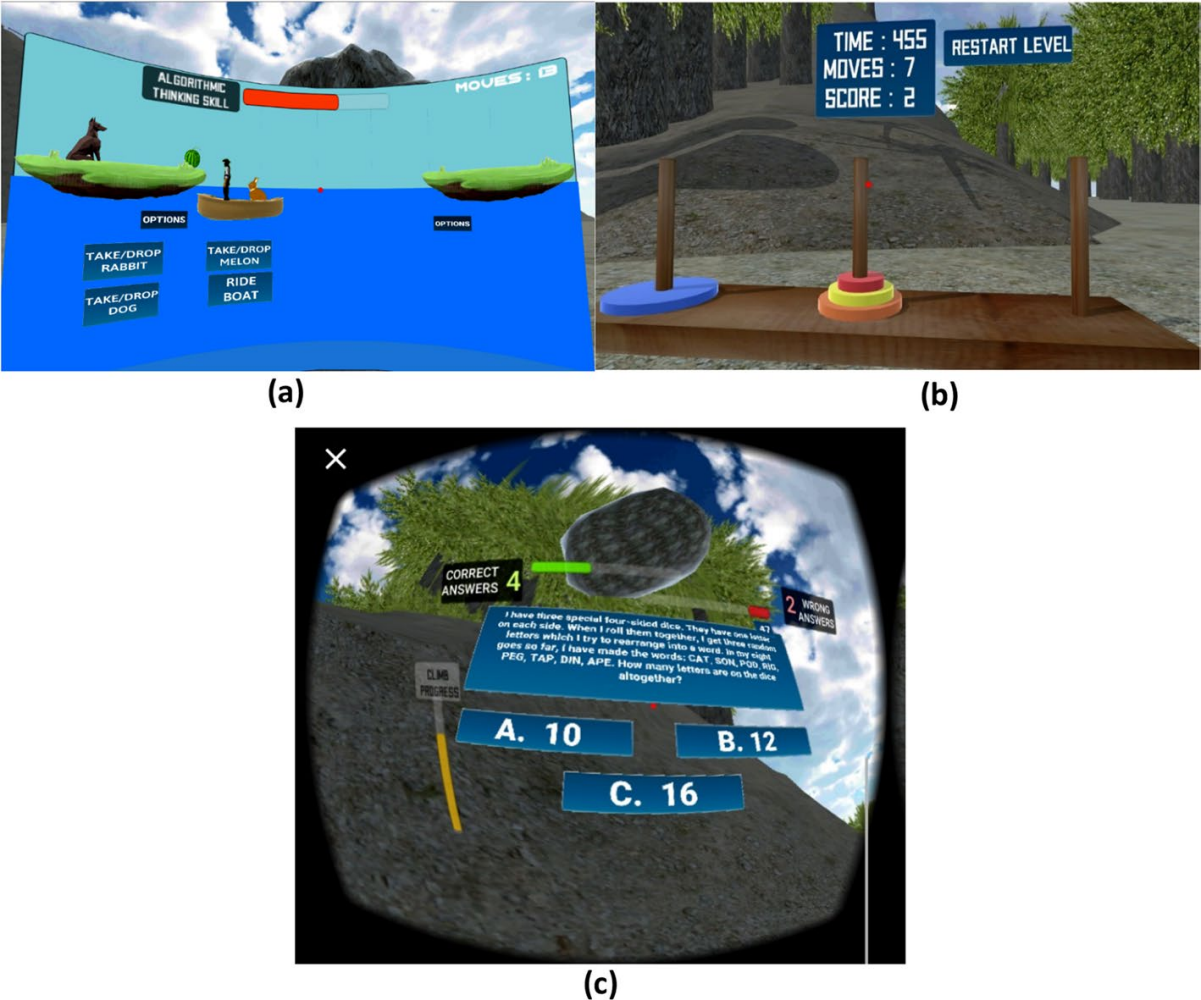
Screenshot showing the iThinkSmart VR mini games: **a** river crossing; **b** tower of Hanoi challenge; **c** MoPaTH

To examine the effect of IVR mini games on higher education learners’ perceived cognitive outcomes, this study experimented with an VR application (iThinkSmart) containing mini games aimed at fostering students’ understanding of computational thinking concepts and supporting players to gain problem-solving skills. In other words, student's computational thinking competencies such as algorithmic thinking, recursive thinking, problem decomposition, abstraction, and pattern recognition can be supported by playing the mini games contained in the iThinkSmart IVR application. These mini games include (a) River Crossing, (b) Mount Patti Treasure Hunt (MoPaTH), and (c) Tower of Hanoi. Figures 3a–c shows the screenshot of these mini games interface.

*The river crossing mini game*, for example, is a logic puzzle that allows students to perform sequential and computational movements of objects while following certain conditions to gain analytical thinking, algorithmic analysis, and problem-solving skills (Lamagna, 2017). In addition, research shows how River Crossing puzzle can demonstrate an AI approach to solving the Breadth-first search (BFS) algorithm (Ratnadewi et al., 2018), which is important knowledge in programming. For example, the IVR app showcased in this study allows students to solve the River Crossing puzzle by applying computational concepts, such as reverse engineering, combination, and algorithmic thinking skills to move items across the river with predefined constraints. By doing so, students can demonstrate how to apply computational thinking concepts to unravel optimization problems. Moreover, finding an optimal solution to an optimization problem remains a critical problem to deal with in computer science.

*The MoPaTH mini game* contains challenges which are computational thinking logic puzzles adapted from Bebras Computing Challenge (2022) and the Beginners Computational Thinking Test (BCTt) (Zapata-Cáceres et al., 2020). The puzzles are presented as multiple-choice quizzes that require critical thinking and problem-solving skills for a player to resolve within the constraint of sixty seconds to unlock a hidden treasure. The MoPaTH mini game simulates a succession of fallen rocks that are directed at the player. Concurrently, there are computational thinking quizzes that display at intervals (typically every one and a half minutes). The player must provide a correct solution to the puzzle in order to stop the rock from falling on him/her. Otherwise, the player suffers from being crushed by the rock and no score point is earned. For every correct solution to a quiz, the player earns a score point and progresses in the climbing of the mountain to the top. Playing the MoPaTH mini game allow students to gain computational thinking and problem-solving skills by, for example, using random outcomes from rolling dice to backward engineer what is contained in their faces, which is one of the BCTt quizzes. The dice problem is common in teaching if-else-if conditional block statements in programming. Therefore, students can gain computational thinking that can lead to their programming knowledge by playing the MoPaTH mini game. Further, the MoPaTH mini game also facilitates the players' expedition experience of a contextual virtual environment.

*The tower of Hanoi mini game* is a mathematical puzzle that contains three pegs and several discs. This puzzle is well known and used in the field of cognitive psychology and mathematics to teach several concepts (Klavžar & Milutinović, 1997; Kotovsky et al, 1985). Because of the connection of these fields to computer science, Tower of Hanoi puzzle is also used to teach computing concepts including recursion (Butgereit, 2016). In the IVR application showcased in this study, the Tower of Hanoi was integrated to visualize recursion so that players can comprehend and gain recursive thinking skills, which is one of the concepts of computational thinking. Recursion is a computational thinking concept that computer science students need to demonstrate programming skills, however, understanding recursion can be difficult, which why this mini game aim to foster its understanding through a visualization approach.

## Research context, ethics, participants, and procedure

This study was conducted at a federal university located in the North-central region of Nigeria. After receiving approval from the institutional head, purposive convenience samples of 60 students were invited to participate in the study. Out of 60 students who registered to participate in the study, 49 students consisting of 38 (77.6%) males and 11 (22.4%) females completed the activities. The number of students that participated in the study was logistically manageable and considered adequate for an evaluation of a VR application as shown in previous studies (Butt et al., 2018; Kim et al., 2020; Lui et al., 2020). Furthermore, physical contact was limited during the time the study was conducted due to the COVID-19 pandemic. Inclusion into the study was intentionally limited to computer science (CS) students because they may have completed CS courses that could give them initial background on computational thinking concepts. The participants gave their informed consent through an online recruitment form where we introduced the goal of the experiment, and informed them that participating in the study was voluntary and that they could withdraw from participating at any stage. In addition, the participants gave their consent to use data collected during the experiment, including the images, for research purposes.

**Fig. 4:**
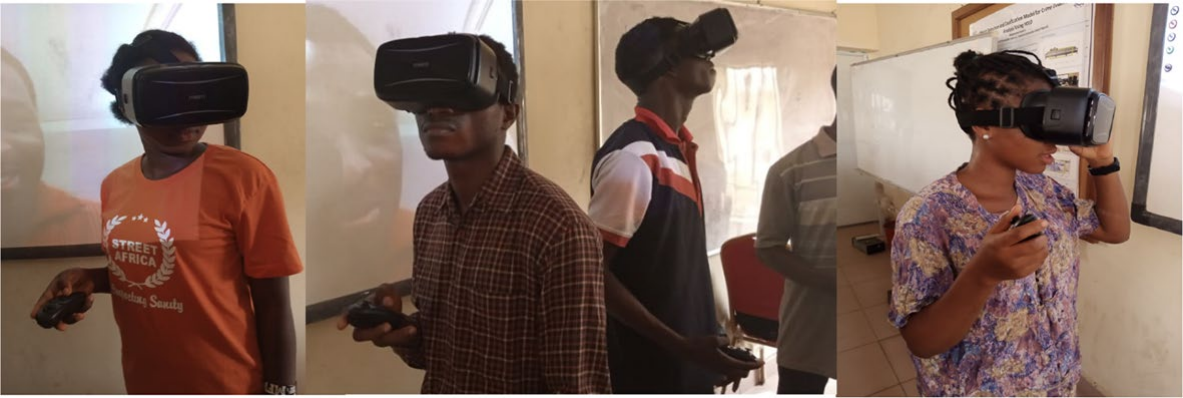
Images showing how participants were engaged in playing the VR mini games

During the experiment, the participants were first introduced to the concepts of computational thinking, HMDs, and how to set up the VR application. This initial set up for the experiment took 30 min. Next, the researchers shared the iThinkSmart VR application for Android Package (APK) with all participants through the Google drive link where they downloaded and installed the App on their smartphones. After the installation was completed, each student played the mini games. The duration for completing the games varied between participants. An average of 30 to 40 min was used for playing the IVR mini games using the HMDs and hand controllers provided by the researchers as shown in Fig. 4. Next, each player completed a survey containing a questionnaire to elicit information on their gameplay and VR experience (described in the next section).

### Instruments

To examine how the IVR features and game elements of an educational tool influence learners’ reflective thinking and comprehension, this study adapted instruments from existing literature. For immersion, we adapted instruments that measure the participants' experience from Hamari et al., (2016) and Makransky and Petersen (2019). For indicators that measure interaction, this study adapted instruments from Bellur and Sundar (2017). Furthermore, instruments to measure participants' experience of game element features (challenge, goal clarity, and feedback) were adapted from Fu et al. (2009) and Fokides et al. (2019), whereas the moderating construct (adequacy of learning contents) instruments were also adapted from Fokides et al. (2019). Additionally, the overall experience of the participants after the experiment was collected using an online form. The analysis of this qualitative data regarding the user’s experience is presented in the result section.

To measure the participant's perceived cognition after playing the mini game, the study adapted the instruments utilized in previous studies on IVR structural model (Lee et al., 2010; Makransky & Petersen, 2019). Although the learning outcomes of the structural model formulated for these previous studies differs from our study, we collected the IVR features and game elements respectively, which forms the independent constructs relevant to this study in examining their effects on learner’s cognition. All the items for the constructs in the structural model were measured with a 5-point Likert scale as shown in Appendix.

### Data analysis

To test the hypothesis formulated in this study and to examine how the IVR features, and game elements of an educational tool influence learners’ reflective thinking and cognition demonstrated in Figs. 1 and 2, partial least squares structural equation modelling (PLS-SEM) analysis was conducted using the WarpPLS 7.0 software (Kock, 2020). PLS-SEM technique is increasingly used in IVR studies (Cheng & Tsai, 2020; Lin et al., 2020; Barrett et al., 2021), whereas WarpPLS software is suitable for examining both factor-based SEM and non-leaner analysis of structurally linked variables in path models (Kock, 2020).

## Results

### Measurement model

This study assessed the measurement models based on the significance of each estimated coefficient or loading, convergent validity, and discriminant validity. We also assessed model fit as shown in Table 1, which demonstrates that the model measurement is satisfied based on the criteria for conducting SEM with WarpPLS (Kock, 2020).

**Table 1:** Model fit and quality indices

No	Quality indices	Criterion	Result	Interpretation
1	Average path coefficient (APC)	*P* value ≤ α (5%)	APC = 0.37, *P* = 0.001	Acceptable
2	Average R-squared (ARS)	*P* value ≤ α (5%)	ARC = 0.46, *P* < 0.001	Acceptable
3	Average adjusted R-squared (AARS)	*P* value ≤ α (5%)	AARS = 0.43, *P* < 0.001	Acceptable
4	Average block VIF (AVIF)	Acceptable if ≤ 5, ideally ≤ 3.3	AVIF = 1.51	Acceptable
5	Average full collinearity VIF (AFVIF)	Acceptable if ≤ 5, ideally ≤ 3.3	AFVIF = 3.44	Acceptable
6	Tenenhaus GoF (FoF)	Small ≥ 0.1, Medium ≥ 0.25,	GoF = 0.59	Large
		Large ≥ 0.36		
7	Sympson's paradox ratio (SPR)	Acceptable if ≥ 0.7, ideally = 1	SPR = 0.88	Acceptable
8	R-squared contribution ratio (RSCR)	Acceptable if > = 0.9, ideally = 1	RSCR = 0.99	Acceptable
9	Statistical suppression ratio (SSR)	Acceptable if ≥ 0.7	SSR = 0.88	Acceptable
10	NLBCDR	Acceptable if ≥ 0.7	NLBCDR = 1	Acceptable

The convergent validity was assessed based on the composite reliability coefficient (CRC) whose value should not be less than 0.7; Cronbach’s alpha coefficient (CRC) whose value should be preferably above 0.7; and average variance extracted (AVE) whose value is recommended to be 0.5 and above according to literature (Hair et al., 2011). On the other hand, the assessment of the discriminant validity was based on the correlations among the latent constructs whose values should be less than the square of AVE (Lin et al., 2020).

Based on the item combined loading in Table 2, all indicators load significantly on their latent constructs with values higher than the recommended thresholds whereas the convergent validity of all constructs meets the requirements as shown in Table 3.

**Table 2:** Item combined loadings and cross loadings

	IMM	INT	CHA	FB	COMP	RFL	GCL	ADQ
IMM1	**0.747**							
IMM2	**0.754**							
IMM3	**0.871**							
IMM4	**0.881**							
INT1	− 0.481	**0.752**						
INT2	− 0.035	**0.781**						
INT3	0.41	**0.880**						
INT4	0.039	**0.733**						
CHA1	− 0.066	− 0.193	**0.874**					
CHA2	0.26	− 0.025	**0.781**					
CHA3	0.191	0.209	**0.967**					
CHA4	− 0.424	− 0.017	**0.778**					
FB1	− 0.247	− 0.375	− 0.51	**0.797**				
FB2	0.494	0.14	0.749	**0.829**				
FB3	− 0.234	− 0.181	− 0.396	**0.706**				
FB4	− 0.419	0.353	0.325	**0.711**				
FB5	0.262	0.062	− 0.173	**0.955**				
COMP1	− 0.064	− 0.021	− 0.053	0.25	**0.936**			
COMP2	0.064	0.021	0.053	− 0.25	**0.936**			
RFL1	− 0.649	− 0.243	− 0.184	− 0.266	0.52	**0.876**		
RFL2	0.377	0.258	0.332	0.047	− 0.791	**0.798**		
RFL3	0.307	0.008	− 0.12	0.225	0.203	**0.868**		
GCL1	0.062	0.078	0.464	− 0.799	0.016	0.109	**0.761**	
GCL2	0.191	0.069	0.39	− 0.089	0.01	− 0.546	**0.847**	
GCL3	0.358	0.07	0.012	0.114	− 0.724	0.339	**0.775**	
GCL4	− 0.66	− 0.233	− 0.941	0.807	0.733	0.159	**0.738**	
ADQ1	0.01	0.007	− 0.019	− 0.009	0.259	− 0.088	0.015	**0.972**
ADQ2	− 0.01	− 0.007	0.019	0.009	− 0.259	0.088	− 0.015	**0.972**

*IMM* immersion, *INT* interaction, *CHA* game challenge, *FB* feedback, *COMP* Comprehension, *RFL* reflective thinking, *GCL* goal clarity, *ADQ* adequate learning contents. Bold values indicates the corresponding latent variables

**Table 3:** Composite reliability, Cronbach Alpha reliability, and AVE

Latent variable	CRC	CAC	AVE
IMM	0.888	0.830	0.666
INT	0.867	0.795	0.622
CHA	0.914	0.872	0.729
FB	0.901	0.859	0.648
COMP	0.934	0.858	0.876
RFL	0.885	0.804	0.719
GCL	0.862	0.786	0.611
ADQ	0.971	0.941	0.944

*CRC* Composite reliability coefficients, *CAC* Cronbach’s alpha coefficients, *AVE* Average variances extracted

Furthermore, the result of the discriminant validity presented in Table 4 shows that the square root of AVE for each construct examined (bold text values in diagonal) is higher than the correlation coefficients for other constructs. The results of the convergent validity and discriminant validity obtained from the SEM-PLS analysis confirmed that the adapted items in the scale were reliable and valid for examining the relationship between game elements and VR features and their impact on learners’ cognition within the theoretical framework of TML.

**Table 4:** Correlations among latent variables with AVEs

	IMM	INT	CHA	FB	COMP	RFL	GCL	ADQ
IMM	**0.816**							
INT	− 0.288	**0.788**						
CHA	− 0.206	0.122	**0.854**					
FB	− 0.111	− 0.01	0.722	**0.805**				
COMP	0.433	0.29	0.135	0.076	**0.936**			
RFL	0.16	0.128	0.316	0.352	0.274	**0.848**		
GCL	0.201	− 0.089	0.681	0.722	0.113	0.512	**0.782**	
ADQ	0.148	− 0.046	0.246	0.511	0.166	0.829	0.545	**0.972**

Square roots of average variances extracted (AVEs) shown on the diagonal; corresponding latent variables are in bold

### The structural model

**Fig. 5:**
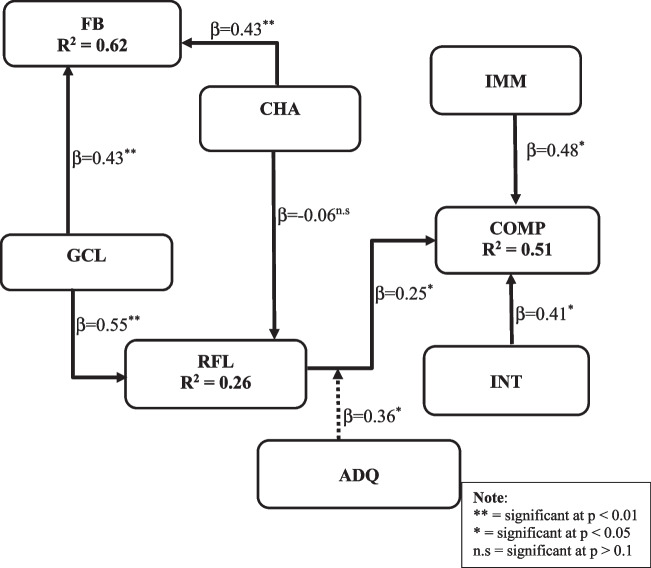
Structural model results

The analysis of the structural model, the relationship between the constructs, and their statistical significance are presented in Fig. 5. The model consists of two parameters, i.e., path coefficient (β) and square multiple correlations (R2). While the path coefficient examines the causal effect of a variable on another connected variable, R2 explains the extent of variation of a construct by the independent constructs (Lin et al., 2020).

As shown in Fig. 5, seven out of eight path relations were found significant with path coefficient (β) ranging from (0.25 to 0.55), thus resulting in seven hypotheses supported and one not supported. Indeed, the result revealed that IMM → COMP (β = 0.48, *P* < 0.01) and INT → COMP (β = 0.41, *P*  < 0.01) have direct, strong, and significant relationships, respectively. Similarly, GCL has a positive and significant direct influence on RFL (β = 0.55, *P* < 0.01); GCL significantly predicts FB (β = 0.43, *P* < 0.01); CHA has a strong and positive influence on FB (β = 0.43, *P*  < 0.01), however, CHA does not positively influence players’ RFL (β = -0.06, *P* = 0.33).

### Moderation

To understand the moderating effect of learning contents deployed in an educational IVR game-based application, this study examined the indirect effect of adequate learning contents among the latent variables of the structural model. In other words, a moderator analysis was conducted to examine the indirect effect of adequate learning content on learners’ cognition by connecting game elements constructs fully mediated by RFL and moderated with ADQ. The moderation result shows that ADQ fully moderated RFL with a positive and significant effect on COMP (β = 0.36, *P* < 0.01).

Therefore, for the direct path-coefficient, immersion shows to be the strongest predictor of learners’ comprehension, whereas, in the indirect path-coefficient, goal clarity remains the strongest predictor of comprehension, respectively. In general, the proposed hypothesis (H1, H2, H3, H5, H6, H7, and H8) were supported while H4 was not supported, as shown in Table 5.

**Table 5:** Standardized path coefficient for tested model

Hypotheses	Path Links	β	T Ratio	*P*-value	Result	Hypothesis results
H1	CHA → FB	0.43	3.55	< 0.01	Significant	Accept
H2	GCL → FB	0.43	3.55	< 0.01	Significant	Accept
H3	GCL → RFL	0.55	4.81	< 0.01	Significant	Accept
H4	CHA → RFL	− 0.06	− 0.45	= 0.33	Not significant	Reject
H5	IMM → COMP	0.48	4.00	< 0.01	Significant	Accept
H6	INT → COMP	0.41	3.34	< 0.01	Significant	Accept
H7	RFL → COMP	0.25	1.94	= 0.03	Significant	Accept
H8	ADQ*RFL → COMP	0.36	2.90	< 0.01	Significant	Accept

T-ratios greater than 1.65 (in absolute value) suggest that the coefficient is statistically significantly different from 0 at the 95% confidence level

Regarding the values of the R2 of the structural model, Fig. 5 presents that the latent variables of educational game elements accounted for 62% of the variance in FB (R2 = 0.62), whereas that of the cognitive factor accounted for 26% of the variance in RFL (R2 = 0.26) and 51% of the variance in COMP (R2 = 0.51).

### Qualitative analysis

The qualitative analysis of the data obtained from participants in this study through open-ended form was analy4sed following the thematic coding approach, a variation entitled “structured tabular thematic analysis (ST-TA)” by Robinson (2022). The ST-TA was developed as a technique for thematic analyses when working with brief qualitative text. In Table 6, we present a summary of codes generated from the open-ended data collected from a few participants. Although the collection of users' feedback was very important to the researchers, the majority of the participant did not feel obliged to fill out the form since the field was intentionally made not compulsory. Notwithstanding, the analysis of the few data provides insights regarding users' experience in interacting with the IVR showcased in this study.

**Table 6:**
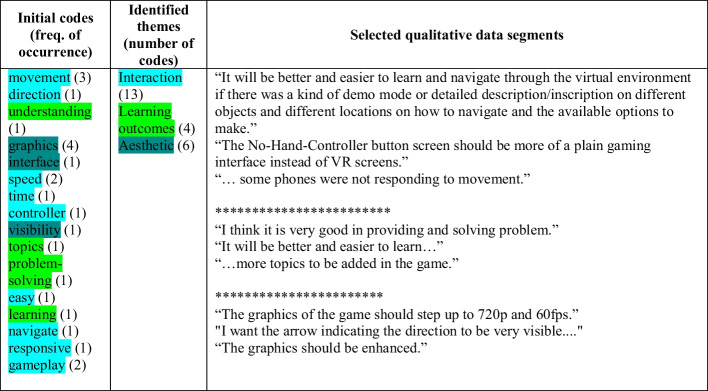
Thematic analysis of participants’ brief qualitative data regarding their experience using the IVR application

**Fig. 6:**
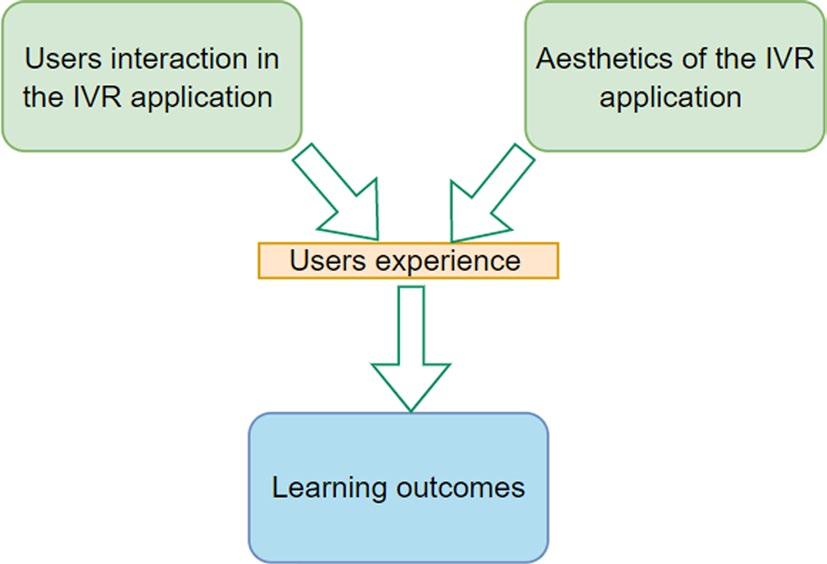
Thematic map of the qualitative analysis of users’ experiences with the IVR application

First, the data was codded, and thereafter, the codes were grouped into themes. Generally, three themes emerged from the codes which include interaction, aesthetic, and learning outcomes. Figure 6 shows a map of themes developed in this study regarding how users’ interaction with an IVR application with its aesthetical characteristics impacts their perceived learning outcome.

Although users were immersed in using the VR application, the overall feedback shows that further improvement is needed to increase the experience. In addition, the result shows that because some aesthetics features in the VR application limit users' experience, their learning outcomes did not seem to be significantly impacted. One possible reason could be that users were distracted by these limitations that the aesthetic features post such that they may not maximize the educational element of the mini games. It is not clear whether the learning content adds to the cognitive load that may affect the learning outcomes, which is contrary to the findings of Makransky et al. (2019).

## Discussion

This study explored how IVR features and educational game elements influence learner's reflective thinking and cognition using an SEM-PLS analysis. The analysis of the measurement model and the structural model was satisfied, indicating that the proposed model was reliable based on all the quality criteria employed. The results indicate several factors that directly and indirectly relate to the learners' perceived cognition using IVR and GBL approaches. In the structural model, feedback, goal clarity, and game challenge have an indirect relationship with learners' comprehension, while immersion and interaction have a direct relationship with learners' comprehension. This study shows the centrality of reflective thinking in the cognition model. Recent research has emphasized the importance of reflective thinking and identified that engaging learners in active reflection need further investigation (Chen et al., 2019). When the goal of learning through a game-based approach is clear to the learners, it can lead to more impactful feedback and improve the experience of playing VR mini games. Similarly, the level of learner engagement through the challenge presented in VR mini games may potentially enhance learners' reflective thinking and consequently influence their comprehension. Surprisingly, this study shows that game challenges did not predict the learner's reflective thinking. As an immersive IVR mini game, one could explain this finding by perceiving that players could find it overloading when the game challenge is increasing. Moreover, Chen et al. (2019) posit that learner's engagement in reflective thinking can generally be a daunting task.

This study differs from the earlier studies conducted by Lee et al. (2010) and Makransky and Petersen (2019), where the authors used usability and VR features to predict reflective thinking. Whereas in this study, the interrelationships of feedback, goal clarity, and game challenge are the indirect determinants of learners’ cognition. Despite the non-significance of game challenge relationship with reflective thinking, it contributes indirectly to the learner's cognition through feedback.

Furthermore, the level of immersion, which could be low or high, and interaction in educational VR applications directly predict the learner's comprehension without any third variable intervention. This study indicates that the higher the learner's immersion and interaction experience with VR mini game, the higher the learner's comprehension. A recent study corroborates the significance of immersion and interaction as features of VR technology on learning experience and learning outcomes (Makransky et al., 2020). While comparing learning through IVR and video, Makransky and his colleagues concluded that the distinguishing mark between IVR and video learning is interactivity, immersion, or a combination of the two factors. The finding from this study regarding how IVR features influence learners' cognition aligns with the findings of Lee et al. (2010), which revealed that immersion of a VR application significantly predicted cognitive benefits. On the contrary, Hamari et al. (2016) argued that immersion did not predict perceived learning outcomes in a non-VR GBL environment. In their study, Cheng and Tsai (2020) highlighted the importance of IVR and its characteristic features in science education, noting that the type of IVR devices and applications can influence users' experience and learning outcomes. Although our qualitative analysis revealed a similar result where certain aesthetic features of the VR application were below users’ expectations and in turn, did not seem to significantly impact their learning outcomes, further research may be required to better understand the conditions under which immersion and interaction influence learner experiences and learning outcomes.

Among the indirect variables, this study revealed that goal clarity is the strongest predictor of reflective thinking, suggesting that the clearer the goal of an educational mini game, the more players would reflect and demonstrate critical thinking. Similarly, the result revealed that the higher the learner's perception of goal clarity, the greater the learner's positive feedback, while the feedback grows with VR mini games challenges. This development indicates that the feedback received from VR mini games based on goal clarity may be positive and rewarding, which in turn may increase motivation for the learners to resolve more challenges.

This study also established the interaction effects of the adequacy of learning contents. The findings show that the interaction effects of reflective thinking on comprehension can be positively influenced by the value of the adequacy of learning content. The interaction effect of the adequacy of learning contents reveals higher relationship strength than when reflective thinking relates directly to learners' comprehension. Interestingly, the adequacy of learning content positively moderates reflective thinking and significantly affects learners' comprehension while playing IVR mini games. Unlike the moderating interaction effects of this study, Cheng and Tsai (2020) earlier used immersion to mediate the relationship between learning traits and learning attitudes, whereas our study delineates immersion as a predictor of the dependent variable (comprehension).

### Limitations of the study

This study involved some limitations. First, there is a limitation due to the small sample size recruited to conduct this study. Future studies could increase the number of participants to obtain more data that may impact the study findings. Second, the study setting was limited to a university and a single department. Widening the scope of the study in terms of settings and disciplines could provide more insightful outcomes. Third, this study only developed its structural model around two independent variables of IVR (immersion and interaction) and three game elements (challenge, goal clarity, and feedback) to investigate the impact of IVR and GBL on higher education learners’ cognition. Future studies could consider including other IVR features and game elements to understand the behaviours of the phenomenon examined. Another important limitation is that all constructs, including learners’ cognition, were measured by student self-ratings, which may be inaccurate. Future research could utilise other measures of cognition, such as performance testing. Although these limitations exist, findings from this study provide an understanding of how an IVR and GBL application can impact learners' reflective thinking and comprehension through a structural modelling approach. In addition, the qualitative analyses provide further insights to reinforce the study’s findings. The implication of this study demonstrates how game elements and IVR features can support the design of an educational intervention to support higher education students' computational thinking skills, particularly in the context of developing countries.

## Conclusions and implications

This study responds to the research gap in IVR literature by showing direct and indirect factors that influence learners' cognition using GBL within the context of higher education students. Also, the study shows the moderating effect of the adequacy of learning contents as a catalyst of learners' cognitive benefits. It is enthralling that the insights from this study show how game elements are associated with IVR features to foster learners' reflective thinking and cognition through engagement, interactivity, and immersion when playing IVR mini games. This finding will help the VR researchers, managers, and educational game developers—particularly from developing countries—to adopt the best practices related to developing IVR game-based applications to foster critical thinking required of all 21st-century learners.

Computer science educators need to pay close attention to learning content through IVR mini games and make it adequate and robust for the students to gain computational thinking competence because it can greatly influence students' reflective thinking and comprehension. Since the virtual environment is an artificial environment capable of improving the learning experience, educators need to stimulate learners' curiosity through reflective thinking. In addition, educators and practitioners should pay attention to the factors responsible for learners' reflective thinking and cognition, such as feedback, goal clarity, game challenge, immersion, and interaction. Practitioners should also consider the moderating strength of adequacy of learning contents between reflective thinking and comprehension. For the learners to maximize cognitive benefits, all six factors (feedback, goal clarity, game challenge, immersion, interaction, and adequacy of learning content) should be considered while planning to design educational IVR game-based applications.

## Data Availability

The datasets generated during this study are available from the corresponding author.

## Appendix 1

See Table 7.

**Table 7:** Instruments used to collect large data on iThinkSmart App

Constructs	Code	Sources	Items
Goal Clarity	GCL1	Fu et al. (2009), Fokides et al. (2019)	Overall game goals were presented in the beginning of the game
	GCL2		Overall game goals were presented clearly
	GCL3		Intermediate goals were presented in the beginning of each scene
	GCL4		Intermediate goals were presented clearly
Challenge	CHA1	Fu et al. (2009)	The challenge is adequate, neither too difficult nor too easy
	CHA2		The game provides ‘‘hints” in text that help me overcome the challenges
	CHA3		The game provides video or audio auxiliaries that help me overcome the challenges
	CHA4		The difficulty of challenges increase as my skills improved
Feedback	FB1	Fu et al. (2009), Fokides et al. (2019)	I receive feedback on my progress in the game
	FB2		I receive immediate feedback on my actions
	FB3		I am notified of new tasks immediately
	FB4		I am notified of new events immediately
	FB5		I receive information on my success (or failure) of intermediate goals immediately
Immersion	IMM1	Hamari et al., (2016), Makransky and Petersen (2019)	I lost track of time while playing the VR application
	IMM2		I became very involved in the VR application forgetting about other things
	IMM3		I was involved in the VR application to the extent that I lost track of time
	IMM4		I forget about time passing while playing the game
Adequacy of the learning contents/material	ADQ1	Fokides et al. (2019)	The good organization of the content helped me to be confident that I would learn this material
	ADQ2		I could relate the content of this game to things I have seen, done, or thought about in my own life
Comprehension	COMP1	Lee et al. (2010)	The VR application made the comprehension of the topic easier
	COMP2		The VR application made the memorization of the topic easier
Reflective thinking	RFL1	Makransky and Petersen (2019)	The VR application enabled me to reflect on how I learned
	RFL2		The VR application enabled me to link new knowledge with previous knowledge and experiences
	RFL3		The VR application enabled me to become a better learner
Interaction	INT1	Bellur and Sundar (2017)	I had the impression that I could be active in the virtual environment
	INT2		I felt that the objects in the VR application could almost be touched
	INT3		I felt the objects in the VR application were aware of my presence
	INT4		When interacting with the virtual objects, these interactions seemed like real ones

## Abbreviations

IVR: Immersive virtual reality
CT: Computational thinking
GBL: Game-based learning
ACM: Association for Computing Machinery
HMD: Head-mounted displays
TML: Technology-mediated learning
FB: Feedback
CHA: Game challenge
IMM: Immersion
GCL: Goal clarity
RFL: Reflective thinking
COMP: Comprehension
ADQ: Adequacy of learning contents
INT: Interaction
VR: Virtual reality
MoPaTH: Mount Patti Treasure Hunt
CS: Computer science
APK: Android package
PLS-SEM: Partial least squares structural equation modelling
CRC: Composite reliability coefficient
CRC: Cronbach’s alpha coefficient
AVE: Average variance extracted
ST-TA: Structured tabular thematic analysis
